# Revisited Metabolic Control and Reprogramming Cancers by Means of the Warburg Effect in Tumor Cells

**DOI:** 10.3390/ijms231710037

**Published:** 2022-09-02

**Authors:** Abekura Fukushi, Hee-Do Kim, Yu-Chan Chang, Cheorl-Ho Kim

**Affiliations:** 1 Department of Biological Sciences, College of Science, Sungkyunkwan University, Seoburo 2066, Suwon 16419, Korea; 2 Department of Biomedicine Imaging and Radiological Science, National Yang Ming Chiao Tung University, Taipei 112, Taiwan; 3 Samsung Advanced Institute of Health Science and Technology (SAIHST), Sungkyunkwan University, Seoul 06351, Korea

**Keywords:** Warburg’s effect, glucose utilization, aerobic glycolysis, metabolic selectivity, cytosolic to mitochondrial pathway determinant, Metabolic enzyme, apoptotic death, carbohydrate metabolic reprogramming

## Abstract

Aerobic glycolysis is an emerging hallmark of many human cancers, as cancer cells are defined as a “metabolically abnormal system”. Carbohydrates are metabolically reprogrammed by its metabolizing and catabolizing enzymes in such abnormal cancer cells. Normal cells acquire their energy from oxidative phosphorylation, while cancer cells acquire their energy from oxidative glycolysis, known as the “Warburg effect”. Energy–metabolic differences are easily found in the growth, invasion, immune escape and anti-tumor drug resistance of cancer cells. The glycolysis pathway is carried out in multiple enzymatic steps and yields two pyruvate molecules from one glucose (Glc) molecule by orchestral reaction of enzymes. Uncontrolled glycolysis or abnormally activated glycolysis is easily observed in the metabolism of cancer cells with enhanced levels of glycolytic proteins and enzymatic activities. In the “Warburg effect”, tumor cells utilize energy supplied from lactic acid-based fermentative glycolysis operated by glycolysis-specific enzymes of hexokinase (HK), keto-HK-A, Glc-6-phosphate isomerase, 6-phosphofructo-2-kinase/fructose-2,6-biphosphatase, phosphofructokinase (PFK), phosphor-Glc isomerase (PGI), fructose-bisphosphate aldolase, phosphoglycerate (PG) kinase (PGK)1, triose phosphate isomerase, PG mutase (PGAM), glyceraldehyde-3-phosphate dehydrogenase, enolase, pyruvate kinase isozyme type M2 (PKM2), pyruvate dehydrogenase (PDH), PDH kinase and lactate dehydrogenase. They are related to glycolytic flux. The key enzymes involved in glycolysis are directly linked to oncogenesis and drug resistance. Among the metabolic enzymes, PKM2, PGK1, HK, keto-HK-A and nucleoside diphosphate kinase also have protein kinase activities. Because glycolysis-generated energy is not enough, the cancer cell-favored glycolysis to produce low ATP level seems to be non-efficient for cancer growth and self-protection. Thus, the Warburg effect is still an attractive phenomenon to understand the metabolic glycolysis favored in cancer. If the basic properties of the Warburg effect, including genetic mutations and signaling shifts are considered, anti-cancer therapeutic targets can be raised. Specific therapeutics targeting metabolic enzymes in aerobic glycolysis and hypoxic microenvironments have been developed to kill tumor cells. The present review deals with the tumor-specific Warburg effect with the revisited viewpoint of recent progress.

## 1. Introduction

In cells, glucose (Glc) is metabolized to acquire energy via the glycolytic and tri-carboxylic acid (TCA) pathways. Cancer cells utilize instead the glycolytic pathway known as an inefficient ATP producing system, generating high amounts of lactate and pyruvate, regardless of the amounts of oxygen molecules in the cells. This distinct phenomenon is called the “Warburg effect” or “aerobic glycolysis”. Since 1920, the phenomenon, indicating that tumors largely utilize Glc molecules, compared to normal cells, had been discovered by the Warburg group [1]. The aerobic glycolysis pathway is a specialized characteristic of tumor cells to acquire energy through Glc uptake and lactate genesis.

In glycolysis steps, certain metabolic enzyme genes such as lactate dehydrogenase (LDH), H^+^/lactate symporters, hexokinase-2 (HK2), keto-HK-A, Glc-6-phosphate isomerase (GPI), 6-phosphofructo-2-kinase (PFK)/fructose-2,6-biphosphatase (PFKFB), glyceraldehyde (GA)-3-phosphate dehydrogenase (GAPDH), phosphor-Glc isomerase (PGI), fructose-bisphosphate aldolase (ALDO), phosphoglycerate (PG) kinase (PGK), triose phosphate isomerase (TPI), and PG mutase-1 (PGAM1), fructose-1,6-bisphosphatase (FBP), pyruvate kinase (PK), PK isozyme M2 (PKM2), enolase (ENO), pyruvate dehydrogenase (PDH) and PDH kinase (PDHK) have been targeted for disruption or silenced to understand the function of the metabolic enzymes [2,3,4,5]. Among them, some enzymes such as PKM2, PGK1, HK, keto-HK-A and nucleoside diphosphate kinase (NME) have dual activities in metabolism and protein kination. For example, both PKM2 and PGK1 protein kinases strongly support the Warburg effect. which can be explained by nuclear PKM2-mediated glycolytic roles and mitochondrial pyruvate shunt of PGK1/PDHK1 axis. The gene expression of these enzymes has representatively been explained by several factors such as hypoxia-induced factor-1 (HIF-1) and c-Myc in glycolytic activation and mitochondrial oxidative inactivation of tumor cells [6].

As the Warburg effect is now accepted in cancer therapeutic study, the glycolysis-inhibitory therapeutics have been considered for targeting tumors. The first exploited agent is 2-deoxy-Glc (2-DG), which it inhibits HK and GPI activities in tumor cells. Others, 2-DG, 3-bromopyruvate, lonidamine, imatinib, resveratrol and apigenin, are known as inhibitors of each glycolytic enzyme [7,8]. However, most of them are under consideration in the preclinical phase. The future discovery and development of metabolic enzyme inhibitors can be arranged for combinatory treatments with other action modes of drugs and with drug resistance. Therefore, the Warburg effect-based cancer therapy has requirements for cancer-specific glycolysis targeting.

## 2. Carbohydrate Metabolic Reprogramming and Adaptation and Direction Decision of Tumor Cell Metabolism

Tumor cells are easily adapted to low oxygen microenvironments that are due to vascular vessel hemodynamics, and metabolically evolve to adapt to the given oxygen environment by low coupled oxidative phosphorylation (OXPHOS) activity in tumor mitochondria, yielding anaerobic glycolysis. Apart from normal cells, tumor metabolic properties decide their proliferating directions, through genetic mutations and the tumor-associate microenvironments [3,9].

To facilitate the antioxidant defense, multiple signaling pathways are involved in tumor cells in their metabolism, such as redox control alterations [3,10]. For example, the well-known PI3K/AKT axis upregulates Glc transport, glycolysis and lipogenesis in tumor cells. Alternatively, the known LKB1/AMPK pathway is indeed a metabolic checkpoint as a metabolic sensor. The LKB-AMPK pathway arrests proliferative behavior under low intracellular ATP/AMP ratios caused by a low nutrient or hypoxia. The axis maintains cellular energy to adapt to metabolic stress before cellular bioenergetic crises such as apoptotic death in a way that AMPK targets mTOR signaling to suppress ACC1 and phosphor-FASN-drive lipid synthesis. Reversely, a hypernutritional condition rather inhibits the LKB-AMPK pathway, contributing to tumorigenic risk in obese or diabetic conditions [11]. During glycolysis, p53 reverses the Warburg effect by inhibition of apoptosis regulator, which decreases a PFK activator known as fructose-1,6-bisphosphatase (F16BP). p53 also upregulates HK and PGAM activities to enhance glycolysis under certain conditions [11,12]. Signaling factors HIF and c-Myc also regulate glycolytic and mitochondrial molecules in tumor cells. For example, c-Myc activates the glycolytic pathway via upregulation of ALDO, ENO, Glc transporters (GluT), GAPDH, HK, PFK, PGI, TPI, PGK, PGAM and PK. In addition, c-Myc increases glutaminolysis level to generate NADPH and mitochondrial level via mitochondrial transcription factor, directing to anabolic metabolism [13]. Similarly, HIF also activates the glycolysis pathway by blocking of pyruvate to mitochondrial entrance. HIF increases ATP genesis and decreases ROS genesis.

## 3. Revisiting the Warburg Effect where Metabolic Enzymes Suppress Tumor Behavior

Some tumor cells such as phaeochromocytoma or paraganglioma exhibit genetic mutations in the mitochondrial succinate dehydrogenase. Furthermore, uterine leiomyoma and renal carcinoma cells exhibit mutations in fumarate hydratase. In addition, gliomas exhibit genetic mutations in NADP+-dependent isocitrate dehydrogenase enzymes [14,15]. Tumor cells metabolically use Glc through the pentose phosphate pathway (PPP) to form NADPH to protect the tumor cells from oxidative ROS environments. In addition, lactate generated from glycolysis enhances tumor behavior to escape from immune surveillance. In fact, aerobic glycolysis generates lactate, causing an acidosis-like pathological condition to confer a better environment for invasive potentials. In addition, the extracellular acidic conditions activate proteases to degrade extracellular matrix (ECM) and suppresses the natural killer and cytotoxic T cells activities. Defected OXPHOS activates ROS genesis to activate HIFs, metastasis and angiogenesis. Among PK isoforms, only PK-M2 isoform as a p-Tyr binding protein is exclusively expressed in cancer cells [16]. PKM2 specifically regulates metabolism and growth by activation of tyrosine kinase (TK) pathways in cancer cells. However, “PKM2 binding partners” inhibit PKM2 by inhibitory phospho-Tyr motifs. In cancer cells, PKM2 itself is Tyr-phosphorylated to be inhibited and PK-M2 modification potentiates switching from OXPHOS to aerobic glycolysis. The TK signaling thus regulates the Warburg effect, although the precise mechanistic explanation on the Warburg effect is not fully understood. Cell surface growth factor receptors with TK activities determine cell metabolism in response to extracellular stimuli [17]. Fibroblast growth factor receptor phosphorylates and inhibits PK-M2 [18]. PKM2 mutants stimulate a metabolic switching from metabolic glycolysis to OXPHOS from glycolysis, attenuating the tumor growth and the Warburg effect. This indicates a prospective insight into TK regulation of tumor metabolism.

## 4. Initial Step of Glc Metabolism in Tumor Metabolic Pathway

**Figure 1:**
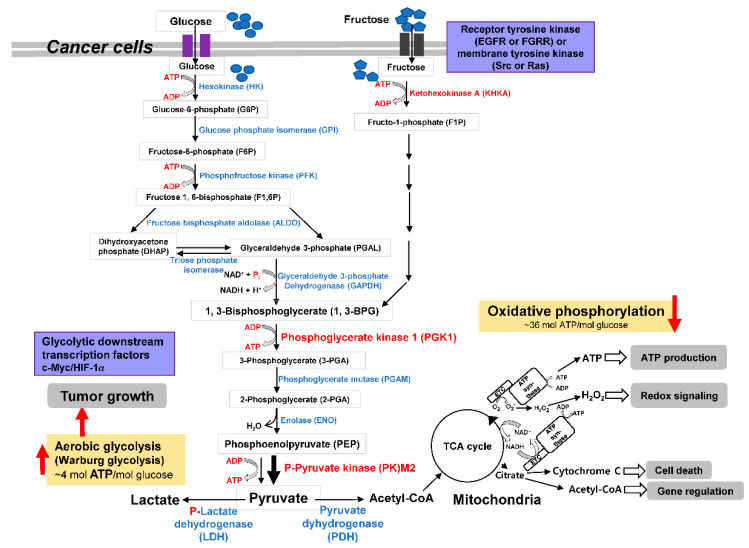
Schematic characterization of metabolic enzymes and metabolites in tumor cells as the Warburg effect. The Warburg effect is revisited to show the aberrant enzyme activity controlled by phosphorylation of metabolic enzymes such as LDH or PDHK.

Glc is a basic supplier of energy and C-backbone (Figure 1). Glc supplies pyruvate in glycolysis to transport to the mitochondria by pyruvate carriers in mitochondria. The mitochondrial pyruvate carrier functions as a cancer down-regulator [19], as the PDH catalyzes the conversion of pyruvate to acetyl-CoA in order to oxidize in TCA cycle. Acetyl-CoA combines with oxaloacetate (OXA) to citrate. OXA in the TCA cycle is transaminated to aspartate (Asp).

## 5. HK Inhibition in Cancer Cells

The first step enzyme in glycolysis is a HK, which phosphorylates Glc to Glc 6-phosphate (G6P) from ATP. Four HK isozymes, HK-1, -2, -3, and -4, are known in mammals. HK-1 and HK-2 are present on the mitochondrial out membrane, HK-3 is in nuclear region, and HK-4 is present in the liver and pancreatic cytoplasmic region. Catalytic activities of HK-1, -2, and -3 are much lower Km values for Glc than the HK-4. HK-2 interacts with voltage-dependent anion channels (VDACs) to acquire mitochondria-generated ATP. HK2 complexed with VDAC is resistant to feedback inhibition of its product G6P. HK-2 easily restores glycolytic substrates. HK-2-VDAC interaction contributed to prevent apoptotic cell death driven by apoptotic Bax in tumor cells [20]. HK1 can phosphorylate histone H2A and HK1 itself. Such phosphorylation is inhibited by Glc [21]. HK1 protein kinase activity shows cellular physiological role. HK-2 with two kinase domains is a cancer-specific enzyme and its expression is enhanced by HIF1 and Myc [22], as a target candidate. The known HK-2 inhibitors of 3-bromopyruvate and benitrobenrazide target the GAPDH to pyruvate the HK-2 protein as the acting target [23]. HK2 is involved in cell proliferation and apoptosis of human cervical cancer cells. Defective HK2 increases the radiosensitive level of cancer cells. HK-2 can bypass a senescence gene expression [24]. HK2 expression activates the hexosamine biosynthetic pathway (HBP) as a glycolytic branch, influencing protein modification via N-/O-glycosylation and O-GlcNAcylation. The HK-2 expression activates some oncogenic molecules such as EGFR, FGFR, AKT, MEK, and β1 integrin [25]. FGF signaling promotes aerobic glycolysis by increasing HK2 expression and the Tyr phosphorylation of aerobic glycolysis. FGF/FGFR1 signaling induces glycolysis and FGFR1 phosphorylates LDHA, where FGFR1 expression induces p-LDHA level and activates LDHA activity in tumor cells. Tyr phosphorylation of LDHA by FGFR1 enhances its tetramerization and NADH binding capacity, consequently increasing the LDHA activity [26]. In comparison, FGFR1 signaling downregulates LDHB, as LDHB function is silenced by hypermethylation of the LDHB promoter [27]. With more precise action, FGFR1 signaling activates aerobic glycolysis by LDHA-Tyr phosphorylation and modulation of LDHB promoter activity. FGFR1-mediated metabolic LDHA activation and LDHB deactivation are associated with cancer growth and progression. LDHA promotes aerobic glycolysis while LDHB facilitates oxidative phosphorylation. This indicates that LDHA is an enhancer of tumor progression by fueling aerobic glycolysis and LDHB is a suppressor in glycolytic tumor cells. FGF signaling also protects from LDHA degradation and deletion of FGFR1 blocks to use lactate as a main energy source and LDHB expression is associated with poor survival in cancer patients.

## 6. Keto-HK Inhibition in Cancer Cells

**Figure 2:**
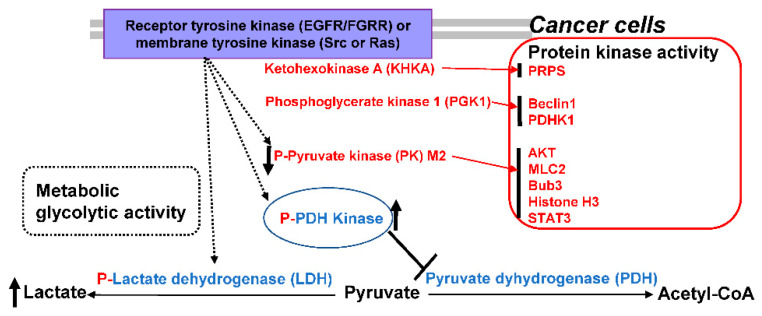
Protein kinase activities of metabolic enzymes PKM2, PGK1, HK and keto-HK-A in aerobic glycolysis.

Fructose metabolism is also important for cell metabolism and mainly regulated by the first-rate limiting enzyme KHK known as fructokinase. KHK converts fructose (Frc) and ATP to its metabolites of Frc1-phosphate (F1P) and ADP, respectively. F1P is further converted to GA and dihydroxyacetone phosphate (DHAP) by aldolase, allowing cooperation with the glycolytic pathway [28]. Exon 3A and 3C forms of the KHK mRNA yield KHK-A and KHK-C. KHK-A enzyme activity is weak in Frc phosphorylation, but KHK-C activity is relatively high due to KHK-C’s high binding affinity for Frc [29]. KHK-A as a protein kinase promotes HCC via nucleic acid biosynthesis (Figure 2). KHK-C is specifically present in the kidney, liver, and pancreas tissues, while KHK-A is widely present. KHK-C enzyme form switches to KHK-A enzyme form in HCC cells, by c-Myc-driven hnRNPH1/2 and KHK-A expressions. KHK-A lowly catabolizes fructose, ATP consumption, and ROS production in HCC cells compared to normal cells. The domain of KHK-A protein kinase binds to the phosphoribosyl pyrophosphate synthetase 1 (PRPS1), a rate-limiting enzyme, rather than fructose to phosphorylate it during the nucleic acid biosynthesis. This phosphorylation promotes proliferation of HCC, supporting KHK-A/PRPS1 phosphorylation axis in HCC progression [28]. Certain enzyme genes such as LDH [4], H^+^/lactate symporters, HK2, keto-HK-A, GPI, PFK/PFKFB, GAPDH, PGI, ALDO, PGK, TPI, and PGAM1, FBP, PK, PKM2, ENO, PDH and PDHK are involved in glycolysis.

## 7. GPI Inhibition in Cancer Cells

Tumor cells are responsive to Glc or insulin to upregulate Glc-utilization. G6P is the key metabolite “repressing” Glc transport activity. In early glycolytic step, GPI functions as a glycolytic and gluconeogenic enzyme, which reversibly isomerize G6P to fructose-6-phosphate (F6P) between the Embden–Meyerhof pathway (glycolysis) and PPP. GPI fluxes G6P into the PPP. GPI disruption diminished Glc usage and lactic acid genesis, affordable for reprogramming of the disrupted cells to follow OXPHOS and mitochondrial ATP genesis. Disruption of GPI, observed in GPI-KO in human colon cancer cells, increases intracellular G6P level [6] and inhibits fermentative glycolysis named the “Warburg effect” in cancer cells. GPI disruption reverts Glc metabolism through OXPHOS in cancer cells. GPI expression is also upregulated by HIF-1 and cMyc in cancer cells [30]. GPI is also an autocrine motility factor involved in metastasis [31]. Disruption of GPI in the tumor cells is not associated with complete growth suppression of tumor cells, but stop rapid growth, due to supplying their energy from both glycolytic and OXPHOS pathways. GPI-KO cells are dependent on oxygen dependent OXPHOS pathway, sensitizing to respiratory inhibitors such as oligomycin or phenformin. Similar results have also been found from the two double KO cells in the LDHA/LDHB genes that completely block glycolytic pathways, but leave a low growth inhibitory effect in tumor cells [6], indicating cancer cell’s metabolic plasticity. Glycolysis-reprogramming blockers for tumor metabolic pathways have been developed at the GPI [10] or LDHA/B inhibitors [32].

## 8. PFK/PFKFB Inhibition in Cancer Cells

In the important step in initial glycolysis event, glycolysis is regulated by multiple enzymes of the PFK and PFKFB [3]. Among the four isoforms, the strongest kinase PFKFB3 generates high Frc-2,6-bisphosphate (F2,6BP) level with glycolysis. Frc-6-phosphate (F6P) is converted to Frc-1,6-bisphosphate (F1,6BP) by a rate-limiting glycolysis enzyme PFK1 regulated by metabolites. PFKFB3 in aerobic glycolysis, generates F2,6BP and F2,6BP acts as an allosteric PFK1 activator. PFK1 as a key modulator of tumor glycolysis converts F6P to F1,6BP [4]. Then, PFKFB in the glycolytic pathway converts F6P to F2,6BP. F2,6BP as an PFK1 activator is generated from F6P by the PFKFB [33]. The dual activities of kinase and phosphatase of PFKFB isoforms determine the net F2,6BP amounts. F2,6BP allosterically activates the PFK-catalyzed glycolysis step. Lactate generated inhibits the PFK1 activity via alteration of the conformational PFK1 structure, while F2,6BP rather stabilizes the PFK1 enzyme in the presence of lactate via allosteric activation and conformational shift [34]. Another metabolite citrate of the TCA cycle also inhibits PFK1 activity [35]. Therefore, the PFKFB3 controls glycolytic level in cells, as its expression involves in dysregulated growth of many cancer cells. The dual kinase and phosphatase activities are present in the PFKFB3 monomer to form homodimers, where the N-terminal domain contains the F6P to F2,6BP converting kinase activity, while the C-terminal domain contains the F2,6BP to F6P converting phosphatase activity [33].

Among PFKFB isozymes, although PFKFB3 is a ubiquitous enzyme in normal cells and tissues, PFKFB3 is overexpressed in invasive tumor cells such as human pancreatic, colon, prostate, and breast cancer cells [36]. PFKFB3 overexpression is associated with glycolytic events to fulfill with the high energy and metabolic requirements for rapid growth, as shown in metastasis of prostate, ovarian, thyroid and lung adenocarcinoma cells [36,37]. In clinical cancers including lung cancers, osteosarcoma, and breast cancer, PFKFB3 expression indicates poor survival rates [37]. Tumorigenic potentials of PFKFB3-expressing colon cancer cells are targeted by its inhibitory agent to regress metastasis [38]. PFKFB3 also induces non-stem cell differentiation to cancer stem cells, depending on the glycolysis [39] and supporting the direct role in rapid cell proliferation and metastasis of cancer cells. The PFKFB3-driven glycolytic pathway is directly associated with angiogenic potentials in endothelial cells. A major PFKFB3 as a cancer-therapeutic target has emerged in rapidly growing cancer cells to regulate metabolic proliferation and several inhibitory agents [40]. Thus, PFKFB3-targetting inhibitors are promising in cancer cells.

## 9. FBP and PKM2 Inhibition in Cancer Cells

FBP induces PKM2 ubiquitination in inhibition of aerobic glycolysis during cancer proliferation. Three enzymes, PK, HK and PFK1, irreversibly catalyze glycolysis. The rate-limiting and allosteric enzyme PFK in glycolysis is enhanced in cancer cells and muscle cells [41]. FBP involves in Glc metabolism by its catalytic action of F1,6BP hydrolysis to F6P and inorganic phosphate [42]. Two vertebral isozymes of FBP1 and FBP2 are known. FBPs are crucial for carcinogenesis. FBP1 expression is specific in kidney and liver, while FBP2 is ubiquitously expressed, where FBP1 and FBP2 share 77% identity of their protein structures [43]. FBP1 and FBP2 are less expressed in gastric cancer due to the promoter methylation [44]. The lowered FBP2 expression in gastric cancer is associated with poor survival and tumorigenesis, as lowly expressed in gastric cancer, triggering glycolysis and proliferation. FBP2 inhibits sarcoma growth by antagonistic action of cytosolic FBP2 to glycolysis, and by restraining action of the nuclear FBP2 to mitochondrial energetic respiration. In cervical cancer, FBP2 expression is inhibited and indicates poor prognosis. FBP2 expression induces regression of aerobic glycolysis, growth and apoptotic death, leading to an anti-tumorigenic potential in cervical cancer [42]. Glycolytic metabolism as a promising subject is attracted in development of therapeutic agent against cancer. Lowly expressed FBP2 negatively regulates aerobic glycolysis and inhibits proliferation of cervical cancer. As FBP2 acts in cervical tumor progression driven by the issued aerobic glycolysis, potentiating cancer aggression and resistance to anti-tumor therapy need to be uncovered.

PK transfers an inorganic phosphate group from phosphoenolpyruvate (PEP) to ADP to generate pyruvate and ATP at the final rate-limiting glycolysis step. The alternatively spliced PKM-type pyruvate kinase (PKM2) form is largely expressed in cancers. Tyr-105 phosphoryl form of PKM2 prevents the active PKM tetramer form in cancer cells. Pro-403/408 hydroxyl form of PKM2 activates HIF1α to enhance the hypoxic upregulation of Warburg metabolic enzymes [45]. The PPP is a key redox metabolic component. PK increases antioxidative levels and its substrate PEP accumulates when PK enzyme activity is decreased, consequently inhibiting the TPI enzyme activity and increasing PPP activity to prevent oxidative stress like ROS. In PKM2 cells, PEP activates the conversion of PGM to a lactate-forming. The TPI inhibition increases the catabolic PPP pathway in glycolysis, as PPP is the NADPH supplier. Cells survive using ATP energy, which is obtained from the metabolic pathway connected to glycolytic fermentation and respiration. Oxidative metabolism efficiently produces ATP but yields ROS. ROS balance is regulated by reduction power of the NADPH. ROS oxidizes cellular compounds such as fatty acids, proteins and others. The reduction power of NADPH regulates ROS balance and also fatty acid synthesis. TPI inhibitors activate the PPP and antioxidative pathway by decreasing ROS levels. Thus, PK/its substrate/TPI axis activates ROS turnover and prevents ROS stress occurred between aerobic and anaerobic shift.

Apart from original metabolic activity in the glycolytic pathway, PKM2 functions as a protein kinase, which is involved in the Warburg effect [46] (Figure 2). In the final glycolytic step, PK activity metabolizes its substrate PEP to convert it to pyruvate, but PEP is also an inhibitor of TPI. PK in glycolysis is crucial and has three alternatively spliced forms of muscle forms PKM1, PKM2 and PKLR (PKR and PKL), where PKLR is specific for red blood cells and the liver. Thus, four isoforms of PKM1, PKM2, PKL and PKR are classified. PKM1 and PKM2 differ in only 22 amino acids sequence. The PKLR gene encodes for PKL and PKR. The PKM gene encodes for PKM1 and PKM2 expressed in different cells. PKM1 switching to PKM2 isoform and PKM2 expression are known in many cancer types [47]. PKM2 has unique nuclear functions, as PKM2 has an exon10-generated nuclear localization signal (NLS), but PKM1 lacks an NLS. ERK/MAPK bind to an PKM2, to form p-Ser37 of PKM2, causing for monomeric conformational shift of a tetramer, and subsequent translocation to nuclear region [48]. Moreover, PKM2 translocation to the nuclear region is stimulated by sumoylation and acetylation activities of PKM2, where Lys433 residues are targeted by the p300 acetyltransferase, consequently preventing the FBP interaction with PKM2 [49]. PKM1 is present in normal cells, while PKM2 is present in tumor cells, although PKM2 is still controversial for its requirement in oncogenesis. Currently, PKM2 is known as the cancer-specific PK isozyme, as its dimeric form has been associated with oncogenesis, acting as cancer-specific signaling molecules such as STAT3, histone H3, OCT-4 and HIF-1 [50,51]. Kinase activity directly phosphorylate histone, as the PKM2 translocates during EGFR signaling and acetylates histone H3, consequently leading to upregulation of proliferation- and progression-related genes [52]. In the nucleus, EGFR signaling leads to PKM2 phosphorylation at Ser37 in the cytosol, but is dephosphated by the Cdc25A phosphatase, allowing a β-catenin complexation and β-catenin binding to its cis elements of MYC and CCND1 genes [53]. Therefore, PKM2 has been targeted to therapeutic approaches for cancer regression. FBP2 induces PKM2 ubiquitination, inhibiting proliferation and aerobic glycolysis in cervical cancers. PKM2 converts PEP to pyruvate and ADP to ATP [54]. Expression of PKM2 as a target of FBP2 is decreased by FBP2 overexpression. PKM2 is highly expressed in cancer cells for cancer growth, progression, glycolysis and chemoradiotherapy resistance [55]. PKM2 overexpression blocks FBP2 action to growth and glycolysis inhibition as well as apoptosis induction. FBP2 blocks cancer progression by PKM2 downregulation. PKM2 is associated with tumor glycolysis and its inhibitor displays anti-cancer effects [56].

Nuclear PKM2 targets phosphorylating reaction of histone H3 at Thr11 using PEP and dissociates histone deacetylase 3 associated with the transcriptional cis-regions to acetylate histone H3 at Lys9 residue. PKM2-phosphorylated histone H3 facilitates EGFR signaling for growth, tumorigenesis and expression of c-Myc-targeted GLUT1 and LDHA genes, increasing PKM2 levels. Therefore, nuclear-translocated PKM2 is involved in glycolysis or the Warburg effect. PKM2 also binds to Oct4, HIF1α, and signal transducer and activator of transcription3 (STAT3) [57]. PKM2′s protein kinase activity facilitates cellular activities. PKM2 phosphorylates Bub3 at Tyr207 and recruits the complex Bub3-Bub1 to Blinkin known as the outer kinetochore protein, promoting chromosomal segregation and growth of tumor cells. Additionally, PKM2 involves in cytokinesis via PKM2 p-Thr45 catalyzed by Aurora B. Some oncogene mutants K-Ras G12V, EGFR variant III, and B-Raf V600E oncogenes enhances PKM2 phosphorylation of myosin light chain 2 (MLC2) Tyr118 [58]. PKM2 expression is enhanced by EGFR-mediated NF-κB and mammalian target of rapamycin complex 1 (mTORC1). PKM2 expression is induced by p-AKT1S1 and mTORC1, and it reduces autophagy in renal and breast cancers [59]. In PKM2 activity, protein kinase enzyme has a phosphorylating activity to Bcl-2 at Thr69 residue, preventing a Cul3-based BCR (BTB-CUL3-RBX1) E3 ligase binding to Bcl-2. This stabilizes Bcl-2 proteins and enhances the oxidative resistance of tumor cells. PKM2 is also known to remodel tumor microenvironments in ECM. PKM2 targets to phosphorylation of synaptosome-associated protein 23, forming the complexed SNARE and extracellularly exosome release [60].

## 10. Fructose-Bisphosphate Aldolase A (ALDOA) Inhibition in Cancer Cells

Among aldolases, ALDOA as a glycolytic and gluconeogenic enzyme receives its prospective attention as a hypoxia-inducible tumor prognostic factor. ALDOA reversibly converts Frc-1,6-bisphosphate (FBP) to DHAP and GA-3-phosphate (PGAL) [61]. FBP is a particular metabolite in cancer, since both gluconeogeneic and glycolytic events require fructose-1,6-bisphosphatase 1 (FBP1) and ALDOA. Increased ALDOA and decreased FBP1 levels are phenotypic characteristics in human cancers without the molecular explanation of how ALDOA and FBP1 regulate the FBP utilization. Defect in FBP1 is associated with poor prognosis of cancer [62], enhancing EMT function, growth and drug resistance of tumor cells [63]. ALDOA regulates FBP levels during glycolysis and the upstream enzyme FBP1 is linked to progression of triple-negative breast cancer cells [64]. As a hypoxia-inducible gene, ALDOA is a HIF1A target in its transcription and ALDOA gene expression is regulated by HIF1α in several tumors such as lung, liver and colon carcinoma cells with resistance and poor prognosis [65,66]. ALDOA expression is directly related to tumor pathology, upregulated by hypoxic conditions, correlated with ROS genesis, glycolytic activity, growth potential, sphere formation, invasion, EMT phenotype, chemoresistance and radioresistance in colon cancer cells [66]. ALDOA also has non-enzymatic functions in invasive potential, drug resistance, and stem cell function of cancer cells [66,67,68]. It has been suggested that the activities of FBP1 and ALDOA are regulated by endogenous proteins during the FBP conversion [69].

Although ALDOA is not associated with sensitivity to chemical agents or radio activities, but seems to be associated with growth and malignant potential even in hypoxic condition, this is attributed to the glycolysis-regulating functions of ALDOA, cell cycle regulation and epithelial-mesenchymal transition (EMT) function. Among the ALDO family, ALDOA involves in the cell cycle by the ALDO binding to F-actin, causing for the M phase defect of cell cycle [70]. Considering the cancer malignancy-associated prognostic hypoxia factors, ALDOA is a therapeutic target, as it is induced by hypoxia in cancer cells and attention is increasing on glycolytic enzymes. Targeting the ALDOA/FBP1 axis has also been considered as treatment to reduce tumor malignancy.

## 11. TPI Inhibition in Cancer Cells

As the 5th enzyme in glycolysis, TPI is essential in cells due to its enzymatic role in Glc metabolism and energy production. The glycolytic enzyme, TPI (EC 5.3.1.1.) is involved in gluconeogenesis, PPP and fatty acid biosynthesis as a metabolic target for its active site-specific inhibitors and tumor-associated marker as well as drug resistance and as a chemotherapy resistance marker in cancers [71]. Homodimer TPI complexes with DHAP as a substrate and isomerizes DHAP to D-GA-3-phosphate (G3P), two hydrolyzed products of F1,6BP by aldolase, during gluconeogenesis and glycolysis [71]. Additionally, TPI provides metabolites to the following metabolic events including the G3P-derived PPP, the quinolinic acid (QA)-derived NAD+ synthesis following Asp oxidization to imino-Asp and DHAP condensation to QA with additional QA conversion to nicotinic mononucleotide/NAD+, gluconeogenesis from pyruvate or oxaloacetate (OAA) and biosynthesis of DHAP-started synthesis of fatty acids and triacylglycerols [71].

TPI deficiency reduces the G3P formation from DHAP and phenotypically shows hemolytic anemia or neuro-retardation [72]. TPI is upregulated in tumor cells and related to patients’ prognosis in various tumor cells including brain, breast, colon, gastric, kidney, lymph node, prostate, renal, testis and urinary cancer cells with metastatic potentials. Especially, TPI enzymes have been regarded as a tumor biomarker of human cancers of gastric cancer and lung squamous cell carcinoma associated with progression and metastasis as well as drug resistance [72,73]. In colorectal cancer, microRNAs of miR-22/-28 enhance the TPI upregulation, reducing lactate levels [73]. TPI is associated with prognosis in gastric cancer, as patients with high TPI expression levels show reduced survival. Thus, TPI is considered to be a potential tumor marker and a new target in gastric cancer treatment. In contrast, TPI1 inhibits liver tumor growth, differently functioning in tumor types [74]. TPI induces expression of epidermal growth factor receptor (EGFR) gene [75]. In hepatocellular carcinoma (HCC), TPI has also been reported to act as a tumor suppressor, which is driven with β-catenin/p53 signaling axis [76]. HCC patients show low levels of TPI expression and TPI upregulation reduces the expression levels of β-catenin and vimentin responsible for EMT. TPI also resists oxidative stress by regulation of glycolysis and PPP. However, there is insufficient information to explain the molecular role of TPI in tumor cells due to heterogeneous tumor types with different phenotypes. In addition, TPI has been suggested to act as an auto-antigen and allergen, and is also associated with neurodegenerative abnormality, calling for a moonlighting protein [77].

On the other hand, naturally, Asn and Gln can be deaminated in proteins, as shown in deamidated human TPI [78] and deamidated TPI has been suggested as a druggable target. Deamidated TPI form accumulates in breast cancer cells but not in normal cells. The deamidated TPI-positive cancer cells are targeted to thiol-reactive drugs-induced apoptosis because they produce a toxic intermediate methylglyoxal (MG) due to DHAP accumulation and also advanced glycation-end products (AGEs) where MG is an AGE-producer [79]. Although MG is converted to a non-toxic metabolite by glyoxalase enzyme, the detoxic conversion of MG is not carried out in oxidative stress conditions. MGO and AGEs are known to trigger apoptotic death. Thiol-reactive drugs can suppress the tumor proliferation because they target the deamidated TIP. Recently, the deamidated TIP has become a new target in breast cancer cells. Deamidated TIP accumulated in cancer cells has been targeted by compounds such as rabeprazole and auranofin to induce apoptotic death and growth inhibition of tumor cells [80]. Therefore, thiol-reactive drugs have been new therapeutic candidates for the cancer therapy and the modified proteins-associated diseases. Currently, an antibiotic ornidazole has been known to inhibit the TPI activity of TPI and caffeic acid phenethyl ester to inhibit the TPI expression in colorectal cancer cells [81]. In a recent study, TPI is nuclear translocated in lung tumorigenesis [82] and its oncogenic role is dependent on nuclear proteins such as Nanog, EZH2 or PCNA to form nuclear complexes. Nuclear translocated TPI enhances drug resistance in tumor cells.

## 12. GAPDH Inhibition in Cancer Cells

As the 6th enzyme in glycolysis, GAPDH converts G3P to D-glycerate 1,3-bisphosphate, present at the first step in NADH genesis, potentiating metabolic flux in the aerobic glycolysis pathway. GAPDH has been attractive as a therapeutic target due to its glycolysis control in the Warburg phenotype of cells such as tumor, activated myeloid and lymphocytic cells [83]. However, the controversial arguments of GAPDH regulation of glycolysis are still unsolved completely yet. GAPDH is frequently expressed in normal cells; however, GAPDH overexpression is crucial for growth of cancer cells. In the glycolysis event of tumor cells, GAPDH is crucial and overall ATP reduction is largely dependent on GAPDH inhibition. This is the reason why GAPDH inhibitors are effective in the regression of tumor cells, which essentially rely on glycolysis for ATP genesis. GAPDH is highly expressed in tumor cells. Targeting and inhibiting GAPDH has been implicated to the cancer treatment. Currently, several GAPDH inhibitors have been known to inhibit GAPDH enzyme activity and proliferation of tumor cells. GAPDH is involved in several diseases. For example, GAPDH forms a complex with huntingtin molecule to cause cytotoxicity in Huntington disease [84]. GAPDH overexpression is characteristic of many tumor cells such as breast, lung, pancreatic, kidney, colon, and esophageal tumor for accelerated proliferation [85]. Hence, silencing GAPDH suppresses the proliferating capacity of tumor cells and causes cancer cell apoptosis.

Glycolysis-targeting agents via GAPDH inhibition should be quantitatively elucidated for the direct correlation between GAPDH and aerobic glycolysis. GAPDH inhibitors are known to covalently bind to the Cys-152 residue of GAPDH catalytic site to inactivate the activity. The catalytic Cys-152 is highly reactive and the nucleophile is reactive to electrophilic agents. From the known GAPDH inhibitors, vitamin C inhibits GAPDH enzyme activity and is lethal to KRAS/BRAF-mutated colon cancer cells [86]. A GAPDH inhibitor, koningic acid also directly inhibits the Warburg effect via inhibiting glycolysis and ATP production, hence inhibiting cell growth [87]. Furthermore, 3-Bromopyruvate (3-BrPA) as a pyruvate analog alkylates thiol groups of GAPDH and other proteins. Several chemicals including DC-5163, which has a 3-(benzyl-N-[3-chloro-4-methoxyphenyl] imidazolidine-1-carbothioamide) structure, Iodoacetate (IA), MG, 2-phenoxynaphthalene-1,4-dione, dimethyl fumarate (DMF), GAPDH segregator, dopamine and 3,4-dihydroxyphenylacetaldehyde (DOPAL) [85,88,89] are also known. DMF binds to the Cys-152 and 4-octyl itaconic recognizes the Cys-22 residue [90]. IA covalently recognizes to the enzymatic active site and NAD+ stabilizes the agent and transition state [91]. Similarly, koningic acid inactivation of GAPDH is enhanced by NAD+ [92]. Thus, the catalytic Cys residue of GAPDH differs from the regular thiol groups, therefore, even low IA concentrations can inhibit GAPDH activity. Interestingly, natural products are also screened the inhibitory activities, as 1,2,3,4,6-penta-O-galloyl-β-D-Glc (PGG) has been isolated as the inhibitory agent, which reversibly inhibits GAPDH activity by a NAD+ and Pi competitive mode [93]. PGG interacts with a NAD+-disrupting and phosphate-binding site, inducing a conformational shift of the tetrameric GAPDH structure. However, such drugs are still limited in use.

## 13. PGK Inhibition in Cancer Cells

Two PGK1 and PGK2 isoforms are known. PGK2 can compensate for the PGK1. PGK1 is a ubiquitous form in all cells while PGK2 is a specific form found in spermatogenic cells and renal, breast, pancreatic, ovarian and testis cancer cells [94]. Only PGK1 with PK can produce ATP during the glycolytic pathway. As the first rate-limiting glycolytic enzyme in ATP genesis, PGK1 reversibly transfers a phosphate from 1,3-diphosphate glycerol ester to ADP, producing ATP and 3-PG, known as a serine precursor [95]. Apart from its phosphatase activity, PGK1 has its kinase activity, as similarly known in other metabolic enzymes such as PKM2, HK, keto-HK-A and NME. PGK1 phosphorylates proteins involved in physiological processes and is widely expressed in human abnormal states such as cancer cells of glioma, breast cancer, and hepatocellular carcinoma [95,96,97]. PGK expression is up-regulated by HIF1 but down-regulated by PPAR-γ [98]. PGK1 acetylation by PCAF and Sirtuin 7 activates glycolysis and tumorigenesis [99]. M2 polarized macrophages secrete IL-6 and phosphorylate PGK1 in glycolysis of tumor cells with malignance and prognosis [100]. However, inhibition of PGK1 does not significantly influence glycolysis. PGK1 silencing increases in DHAP and GA3P levels, enhancing MG production. PGK1 inhibitor CBR-407–1 also increases DHAP and GA3P levels with MG production, decreasing ubiquitination [100]. The results are similar to those of GAPDH silencing and GAPDH inhibitors in DHAP and GA3P levels with MG production. PGK1 silencing also inhibits growth of HeLa cells by the non-glycolysis pathway [101].

Cytosolic PGK1 is translocated to mitochondria by the known signaling of EGFR/K-Ras G12V/B-Raf V600E/ERK axis [102] via Ser-203 residue phosphorylation of PGK1. The mitochondrial PGK1 activates PDHK1 by phosphorylation of Thr-338 residue of PDHK1. The activated PDHK1 phosphorylates Ser-293 residue of PDH E1α, causing PDH complex inactivation, which prevents mitochondrial condensation of CoA and pyruvate to acetyl-CoA and CO2. Therefore, PGK1 activity consequently blocks OXPHOS and produces lactate by shunting mitochondrial pyruvate into the cytoplasm. This eventually helps tumor proliferation [102]. Thus, the Warburg effect can be explained by nuclear PKM2-mediated glycolytic genes such as PKM2 itself for Glc acquisition and lactate genesis and mitochondrial pyruvate shunt of PGK1/PDHK1 axis. During autophagosome formation, Beclin1 recruits the PI3K to generate PI3P, which recruits PI3P–binding domains [103]. Acetylated PGK1 initiates autophagy event by phosphorylation of Beclin1 Ser30 residue, causing for growth and poor prognosis for tumor cells [104]. As a protein kinase enzyme, PGK1 regulates autophagy initiation, mitochondrial function and tumor cell proliferation (Figure 2). The EGFR/ERK axis-driven casein kinase 2α, CK2α, phosphorylates the PGK1 [101], consequently the p-PGK1-bound to CDC7 recruits DNA helicase and promotes cell growth. PGK1 phosphorylation of Beclin1 Ser30 leads to hypoxic tumorigenesis of brain cells [105]. PGK1 also phosphorylate Thr-338 residue of PDHK1, leading to inhibition of PDH complex activity and lactate production with brain tumors. Thus, PGK1 is a promising target candidate for glioma treatment. Among the PGK inhibitors, NG52 inhibits the Thr-338 phosphorylation of PDHK1 and the Ser-293 phosphorylation of PDH, enhancing pyruvate production for the TCA cycle, and ATP and ROS genesis. NG52 [103] as a PGK1 kinase inhibitor enhances PDH activity by PGK1 inhibition in blocking the glioma growth [105]. PGK1 inhibitors are a promising anticancer agent. For example, non-ATP-competitive inhibitor of PGK1 has been discovered from natural products, binding to Cys379 and Cys380 of PGK1. The agent inhibits hypoxic PGK1 and EGF/EGFR signaled protein kinase activity of PGK1 to phosphorylate Thr338 residue of PDHK1 in glioblastoma cells. The drug decreases lactate production and Glc uptake, and subsequently induces apoptotic death of glioblastoma cells, inhibiting the tumor growth [106].

## 14. PGAM Inhibition in Cancer Cells

PGAM exerts metabolic function in Glc metabolism *in vitro*, however, its glycolytic importance in vivo is still unclear. Among the known PGAM-1 and -2, PGAM-1 is deeply involved in cancer metabolic pathway, as deduced from the previous result that homozygous Pgam1 KO mice are lethal at the embryonic state [107]. It critically converts 3-PG to 2-PG in the aerobic glycolysis pathway, simultaneously regulating precursor intermediates to be used for anabolic biosynthesis [108]. PGAM1 is highly expressed in several cancers found in lung, urothelial bladder, non-small-cell lung cancer and HCC [109]. PGAM1 also promotes homologous DNA recombination by metabolic regulation of the nucleotide pool [110]. In contrast, it also potentiates tumor migration by binding to α-smooth muscle actin (SMA) [111]. Therefore, PGAM1 has been potentially targeted as a promising therapeutic candidate for tumor therapy. PGAM converts 3-PG to 2-PG [112]. PGAM consists of two isoforms, PGAM1 and PGAM2 with a high similarity. PGAM1 promotes tumor progression and aggravates tumor cells via activated glycolysis and interaction with SMA [113]. The 2-PG product of PGAM activates the PPP to generate reducing agent NADPH [108]. PGAM inhibits oxidative respiration by mitochondria and thus PGAM reduces level of mitochondrial ROS by the PPP [114]. p53/Mdm2 axis is responsible for PGAM, the ubiquitous degradation in cellular stressed conditions [113] and p53 diminishes glycolysis in cancer. Phosphorylation or acetylation of PGAM reduces its enzyme activity [115], but many cancerous tissues show an increased level of activity. PGAM is regulated in cancer glycolysis. PGAM cooperated with a p53-specific checkpoint kinase, Chk1, to enhance glycolysis in cancer cells. The glycolytic PGAM-Chk1 interaction upregulates cancer behavior [116]. Defected PGAM1 decreases growth level of urothelial bladder cancer cells. Therefore, PGAM1 has been considered as a treatment target of cancers. For example, an allosteric inhibitory agent, HKB99, immobilizes the PGAM1 confirmation to suppress growth and metastasis of tumor cells. HKB99 is also active against EGFR-specific low molecular erlotinib-resistant tumor cells, emphasizing the importance of PGAM1 inhibitors such as HKB99 for a tumor-therapeutic agent [109]. Currently, a limited number of PGAM1 inhibitors are known with MJE3, EGCG, 12r and PGMI-004A [108,117,118,119], although PGAM1 inhibitors are promising anticancer agents. The inhibitory mechanism of PGMI-004A has been explained, as it has been described that it allosterically interacts with PGMA1 to compete with 3-PG [109].

## 15. ENO1 Inhibition in Cancer Cells

Currently, the four metabolic approaches in tumor therapy include inhibition of glycolytic enzymes and PPP enzymes, OXPHOS activation and HIF-1 activity downregulation [120]. ENO converts 2-PG to PEP. Three isozymes of α-enolase, β-enolase, and γ-enolase, abbreviated ENO-1, ENO-3, and ENO-2, respectively, are known with homodimer forms. The enzyme expression has been argued in cancer relevantly for the Warburg effect, which is adaptively responded to hypoxic condition in tumor cells. ENO-1 is also associated with mitochondrial membrane potentials and receptor functions on the cell surface. ENO1 constitutes a protein complex as a multi-functional enzyme with urokinase-type plasminogen activator receptor, integrin and plasminogen receptor on the tumor surfaces, which promotes plasminogen/plasmin conversion, while in the cytoplasm, ENO1 potentiates tumor cell migration, invasion and metastasis [121]. It converts 2-PG to PEP in the final glycolytic step. ENO1 maintains “aerobic glycolysis and can causes autoantibody genesis in cancer patients [122]. Reprogramming of metabolism in cancer cells involves in increased glycolysis to synthesize nucleotides, lipids and proteins required for cancer growth. The ENO1 metabolically maintains the Warburg effect in tumor cells and its inhibition induces reactive oxygen species [123]. ENO1-deficient tumor cells highly uptake Glc, causing for the PPP or the polyol pathway (PP), characteristic for the decreased lactate levels. The ENO1-deficient tumors sensitively respond to the oxidative stress-increased autophagy and acquire the energy from fatty acid oxidation, causing growth retardation [123]. To date, approaches to target ENO1 metabolism are based on the tumor-specific metabolic switches. Tumor metabolic ENO1 has been potentially therapeutic target of tumor behavior. Anti-cancer strategies targeting the Glc metabolic pathway are promising [124,125]. ENO1 is a key glycolytic enzyme, conferring its inhibitory merits as a representative approach. For example, ENO1 enzyme inhibitors currently known include 3-aminoenolpyruvate 2-phosphate, sodium fluoride and D-tartonate, although they are not therapeutically appropriate in use. Interestingly, a pan-enolase transition-state analogue inhibitor, phosphonoacetohydroxamic acid inhibits growth of tumor cells [123]. The analogue inhibitor “ENOblock” (AP-III-a4), inhibits the ENO1 enzyme activity via its direct binding to the enzyme structure and metastatic potential. ENO1-specific antibodies disturb ENO1 binding to plasminogen to inhibit migration and invasion. Increased levels of antibodies against phospho-ENO1 correlate with survival rate in cancer patients [126]. Nuclear-translocated ENO1 activity directly enhance gene expression of targets. The ENO1 interacts with certain DNA sequence to enhance gene expression, as ENO1 protein alternatively splices a 37 kDa mRNA of Myc promoter-binding protein-1 to bind to c-myc P2 promoter to repress its transcription [127]. In conclusion, ENO1 has been promising as a future therapeutic strategy.

## 16. LDH Inhibition in Cancer Cells

Hypoxic condition upregulates LDH activity, an essential enzyme of the Warburg effect, and LDH is present as two different forms of LDHA (homotetramer) and LDHB (heterotetramer). LDHA reduces pyruvate-lactate conversion rate via regeneration of NAD+ from NADH. LDHB oxidizes lactate to pyruvate by tumor cells [128]. LDHA inhibitors are therapeutically a major target to inhibit tumor growth as known in lung cancer and a xenograft transplanted tumor, although LDHB is also a target [129]. The developed LDHA inhibitors, GSK2837808A and GNE-140 are potent with IC50 2 nM [130] and 3 nM, respectively [131]. The allosteric LDHA inhibitors target allosteric pockets, but not catalytic domains, in NAD+/NADH cofactor-binding site.

LDH inhibitors are also being designed and developed, including FX11 and oxamate, although they are not the clinical subjects [132]. LDH inhibitors generally inhibit growth of tumor cells, but not of normal cells, reducing LDH activity, lactate and ATP genesis at hypoxic and normoxic conditions as well as Glc uptake. Intracellular ATP levels are decreased and the uptake of Glc is also reduced with the reduced growth through inhibition of the LDH activity as a key enzyme to regulate tumor metabolism. Epigallocatechin gallate is also a LDH inhibitor [133], as LDH inhibitors inhibit pyruvate conversion to lactate, consequent pyruvates are used in TCA cycle, shifting from glycolysis to aerobic TCA cycle/OXPHOS axis in mitochondria. This metabolically shifted change in energy acquisition raises a demerit to tumor cells, causing for the increased oxygen consumption and ROS genesis. The situation also raises mitochondrial oxidative damage. Although the decreased Glc uptake and ATP levels are not always based on LDH inhibition [134], energy crisis is essential. Thus, LDH inhibitors can lead to the reduced levels of intracellular ATP and Glc uptake.

## 17. PDHK Inhibition in Cancer Cells

In tumor metabolic enzymes, many studies have been focused on PDHK as a target as well as LDH for reducing aerobic glycolysis. Some natural products such as wogonin have been studied as inhibitors of HK, LDH, PK-M2 and PDHK expression as the glycolysis pathway enzymes [135]. PDHK downregulates PDH activity via phosphorylation. However, pyruvate of tumor cells is mainly converted to lactate regardless of the oxygen, not entering mitochondrial cycle. This phenomenon is now concluded by upregulated PDHK activity and downregulated PDH activity. The mammalian PDH complex (PDC), comprising of PDC E1α subunit (PDHA), dihydrolipoamide acetyltransferase (E2) and lipoamide dehydrogenase (E3), decarboxylates pyruvate to acetyl Co-A for the glycolytic to oxidative TCA cycle progression [136]. The PDHK enzymes reduce pyruvate utilization. Mitochondrial PDHK enzymes involve in aerobic glycolysis, where they phosphorylate the PDHA, claiming PDHK as a promising target candidate of cancer regression. PDHK regulates energy production via aerobic glycolysis by PDC inhibition [137]. To transit phosphoryl oxidation to glycolysis, the PDC inhibition occurs via PDHA E1α subunit kination by PDHK [138]. Four PDHK1-4 isoforms are known in humans and PDHK1 is predominantly present in tumor cells in hypoxic environments. The other PDHK2-4 isoforms are also tumor type dependent, but not dominant in the tumor cells [139]. Interestingly, PDHK1 specifically phosphorylates three Ser residues of PDH E1α subunit. The E2′s L2 domain region docks PDHK after PDHK isoforms interact with the PDC E2 subunit to form a phosphor-PDHA at Ser232, Ser293, and Ser300 residues [140]. In hypoxic malignant tumors, a PDHK1 mainly phosphorylates PDHA to block PDC activity in mitochondria, indicating that it is an essential enzyme for the glycolytic pyruvate entrance to TCA cycle in mitochondria. PDHK1 is therefore a promising target in cancer metabolism [141]. Apoptotic induction of PDHK1 inhibitors is seen in tumor cells, but not in normal cells. Thus, it has been suggested that PDHK1 inhibitors differentially influence growth of non-cancer cells and cancer cells have been suggested to be caused by higher mitochondrial respiration in normal cells than malignant cells [142]. PDC directly keeps the OXPHOS status, catalyzing the pyruvate conversion to acetyl-CoA. This is the mitochondrial first step operated in the TCA cycle [143]. PDC is therefore a promising target to inhibit lactic acidosis and cancer growth [144]. Although cancer cells utilize glycolysis more than OXPHOS in normoxic conditions as the Warburg effect, aberrant aerobic glycolysis stimulates PDHK activity. PDHKs increase electron-donor NADH and electron transport chain complex-driven ROS levels in tumor mitochondria. PDHKs inhibit the release of apoptotic factors [145]. For example, dichloroacetate (DCA) analogs such as radicicol recognize the ATP-binding pocket of PDHK to inhibit the PDHK activity [142].

PDHK inhibitors as a cancer-targeting candidate are receiving a great attention from synthetic and natural resources [146,147]. For example, AZD7545, DCA and phenylbutyrate (PB) are those PDHK inhibitors under consideration [142,148]. Among them, limited PDHK inhibitors of DCA and PB are clinically known. For mechanistic action of thee PDHK inhibitors, DCA-inhibition of PDHK1 activity transforms tumor metabolic glycolysis to phosphoryl oxidation status, consequently decreasing the resistant level of mitochondrial apoptosis [140,141]. From natural products, some PDHKs inhibitor are also known, including methyl jasmonate, thiazolidinediones, huzhangoside and shikonin. The inhibitors downregulate glycolysis event such as Glc uptake, HK, and PK [147,148,149,150], inhibiting the Warburg effect.

## 18. Critical View of Anti-Cancer Therapeutics Based on Understanding the Warburg Effect

Given the tumor aerobic glycolysis, metabolic enzymes in the glycolytic pathway are targets for the Warburg effect. Development of glycolytic enzyme inhibitors is a promising antitumor strategy. Currently, a number of anti-cancer therapeutics, targeting anaerobic glycolysis, have been developed from natural and synthetic compounds to target metabolic enzymes [151,152]. However, most of them are mainly investigated in in vitro cell levels and animal models, but not human clinical trials. For example, low molecular weighted inhibitors to target glycolytic enzymes in tumor cells are being developed. Although new metabolic enzymes in tumor cells are also being studied for the development of new enzyme inhibitors [153], aerobic glycolysis and hypoxic microenvironments-targeting therapeutic agents are still considered to kill tumor cells with development of biomarkers.

## 19. Conclusions

The Warburg effect revisited herewith has diversely examined the evidence for related enzymes, carbon source transporters, carbohydrate metabolites, and gene expressions, which would be associated with poor prognostic makers, diagnostic values, progression modulators, and therapeutic inhibitors of cancer. In cancer cells, cancer-borne phosphorylation of metabolic enzymes represents the Warburg effect, as illustrated in Figure 1. Metabolic differences between cancer and normal cells are promising for tumor-targeting strategies. Although the metabolic pathway of tumor is too heterogeneous, reprogramming of pyruvate to lactate formation is the pivotal step in survival of the aggressive and hypoxic cancer cells. The Warburg effect inhibitors inhibit tumor growth through the activity inhibition of metabolic enzymes involved in the glycolytic pathway in tumor cells. Representatively, inhibitors of LDH and PDHK, HK, GPI, PFK, PFKFB, GAPDH, PGI, ALDO, PGK, TPI, PGAM1, FBP, PK, PKM2, ENO, PDH and PDHK are definitely studied with actual inhibition of lactate production by such inhibitors. In parallel, the ROS genesis and mitochondrial damages are also increased by these metabolic inhibitors. Therefore, potential anti-cancer drugs are still desired for development in a way of the shift of metabolic pathway from aerobic glycolytic pathway to Frc or Glc metabolic enzyme inhibitors, targeting those enzymes including LDH and PDHK. Our understanding of carbohydrate metabolism, metabolic adaptation and genetic adaptation to tumorous glycolysis will open a prospective paradigm and vista to control abnormal behavior of transformed cells or tumor cells, if more reasonable evidence is elucidated in the near future.

## Data Availability

Not applicable.
